# Pan-PI3K inhibition with copanlisib overcomes Treg- and *M*2-TAM-mediated immune suppression and promotes anti-tumor immune responses

**DOI:** 10.1007/s10238-023-01227-6

**Published:** 2023-11-08

**Authors:** Simon Heller, Sarah Glaeske, Katja Gluske, Juliane Paul, Annika Böhme, Andreas Janzer, Helge Gottfried Roider, Anna Montebaur, Barbara Nicke, Ralf Lesche, Oliver von Ahsen, Oliver Politz, Ningshu Liu, Mátyás Gorjánácz

**Affiliations:** 1 Nuvisan ICB GmbH, Berlin, Germany; 2 Bayer AG, Pharmaceuticals, Research & Early Development Oncology, Berlin, Germany; 3 Present Address: Fosun Pharma, No. 1289 Yishan Road, Shanghai City, China

**Keywords:** Aliqopa, Copanlisib, Immune checkpoint inhibition, Immune suppression, PI3K pathway, Tumor microenvironment, CD8^+^ T cells, Tumor-associated macrophages, Tregs

## Abstract

The PI3K pathway is one of the most frequently altered signaling pathways in human cancer. In addition to its function in cancer cells, PI3K plays a complex role in modulating anti-tumor immune responses upon immune checkpoint inhibition (ICI). Here, we evaluated the effects of the pan-Class I PI3K inhibitor copanlisib on different immune cell types in vitro and on tumor growth and immune cell infiltration in syngeneic murine cancer models. Intermittent treatment with copanlisib resulted in a strong in vivo anti-tumor efficacy, increased tumor infiltration of activated T cells and macrophages, and increased CD8^+^ T cell/regulatory T cell and *M*1/*M*2 macrophage ratios. The strong in vivo efficacy was at least partially due to immunomodulatory activity of copanlisib, as in vitro these murine cancer cells were resistant to PI3K inhibition. Furthermore, the combination of copanlisib with the ICI antibody anti-PD-1 demonstrated enhanced anti-tumor efficacy in both ICI-sensitive and insensitive syngeneic mouse tumor models. Importantly, in an ICI-sensitive model, combination therapy resulted in complete remission and prevention of tumor recurrence. Thus, the combination of ICIs with PI3K inhibition by intermittently dosed copanlisib represents a promising new strategy to increase sensitivity to ICI therapies and to treat human solid cancers.

**Supplementary Information:**

The online version contains supplementary material available at 10.1007/s10238-023-01227-6.

## Background

Immune checkpoint inhibitors (ICIs), such as programmed cell death receptor 1 (PD-1) antibodies, have shown promising long-lasting effects in several human cancer types via T cell-mediated anti-tumor immunity [1, 2]. The efficacy of ICIs was reported to positively correlate with the density of tumor-infiltrating CD8^+^ T cells [3–6]. However, most solid cancers are characterized by poor CD8^+^ T cell infiltration and do not respond to ICIs [7, 8]. Although the mechanisms involved in the absence of T cells are not completely understood, it has become clear that promoting T cell infiltration may turn non-inflamed “cold” tumors into T cell-inflamed “hot” tumors and broaden the range of cancer types responding to ICIs [9–11]. In addition to low amounts of CD8^+^ T cells, immunosuppressive tumor microenvironments (TMEs) are often characterized by higher proportions of regulatory T cells (Tregs), *M*2 subtype of tumor-associated macrophages (TAMs), and myeloid-derived suppressor cells (MDSCs) [12–17].

The PI3K pathway is one of the most important oncogenic pathways. Its activation promotes cell proliferation, survival, migration, angiogenesis, metabolic reprogramming and contributes to the establishment of an immunosuppressive TME [18–22]. PI3K is also a critical regulator of immune cell functions, especially through the relatively exclusive expression of the isoforms γ and δ in addition to the broadly expressed isoforms α and β [19]. Thus, PI3K inhibitors may cause a beneficial modulation of the immune cells in the TME, in addition to their direct cytotoxic anti-tumor effects [21, 23–25]. Indeed, inhibition of PI3Kδ has been reported to inhibit immunosuppressive Tregs and, in some cases, increase the infiltration and activation of cytotoxic T cells, while other studies have reported negative effects on CD8^+^ T cells [12, 13, 26–28]. A dual PI3Kα/δ inhibitor showed promising modulatory effects on T cells but caused a reduction of intratumoral macrophages and an increase of MDSCs [12, 26, 27, 29]. In contrast, PI3Kγ inhibitors were shown to promote CD8^+^ T cell infiltration and modulate intratumoral macrophages to a pro-inflammatory *M*1 state [30–32].

Furthermore, these studies demonstrated that PI3K inhibition may counteract ICI resistance, resulting in improved anti-tumor efficacy of combination therapies [30–32]. PI3Kδ inhibitors have also shown synergy with ICI but a detrimental effect was reported upon systemic depletion of PI3Kδ [27, 28]. Treatment with pan-PI3K inhibitors caused anti-tumor efficacy in monotherapy and in combination with ICI, despite an unexpected increase of Tregs and a depletion of macrophages [33]. These findings underline the complex interplay of PI3K isoforms in the regulation of intratumoral immune cells. To maximize the beneficial and minimize the disadvantageous effects, the overall outcome of PI3K inhibition on anti-tumor immunity should be carefully investigated.

Copanlisib is an intravenously and intermittently administered pan-Class I PI3K inhibitor which has been approved by FDA for treatment of relapsed follicular lymphoma [34]. Here, we evaluated the effects of copanlisib alone and in combination with anti-PD-1 in two syngeneic murine colorectal cancer models. To distinguish the immunomodulatory from the direct anti-tumor effects, we selected models that were either responsive or resistant to PI3K inhibition in vitro and were differently sensitive to ICIs in vivo. Particularly, we studied the effects of copanlisib on CD8^+^ T cells, Tregs and TAMs, both in vitro and in vivo*,* thereby elucidating the mechanisms that might overcome the immunosuppressive TME.

## Methods

### Reagents and cell lines

Copanlisib was synthesized at Bayer. Idelalisib (CAL-101), alpelisib (BYL-719), TGX-221, and eganelisib (IPI-549) were purchased from Selleck Chemicals (Munich, Germany) and the anti-mouse PD-1 (anti-PD-1, clone RMP1-14, Catalog #6E0146) from Bio X Cell (West Lebanon, New Hampshire, USA).

CT26 and THP-1 cells were obtained from ATCC and MC38 cells from NCI. All cell lines were authenticated by fingerprint techniques and tested for mycoplasma contamination at DSMZ (Braunschweig, Germany). The cell lines were maintained in medium containing 10% heat-inactivated fetal calf serum (FCS) according to the instructions by the providers.

Antibodies for flow cytometry (Table 1) were purchased from BioLegend (San Diego, LA, USA). Antibodies for immunohistochemistry were purchased from various sources (Table 1).

**Table 1:** Antibodies

Antibody	Isotype	Clone	Manufacturer	Cat#
Antihuman CD3	mouse IgG1κ	UCHT1	BioLegend	300401
Alexa Fluor® 647 antihuman CD3 antibody	Mouse IgG1,k	SK7	BioLegend	344825
Alexa Fluor® 488 antihuman CD4 antibody	Mouse IgG1,k	RPA-T4	BioLegend	300519
Pacific Blue™ antihuman CD8 antibody	Mouse IgG1,k	SK1	BioLegend	344718
FITC antihuman CD8 antibody	Mouse IgG1,k	SK1	BioLegend	344703
PerCP/Cyanine5.5 anti-human CD25 antibody	Mouse IgG1,k	M-A251	BioLegend	356112
PE antihuman CD25 antibody	Mouse IgG1,k	BC96	BioLegend	302605
PE antihuman CD25 antibody	Mouse IgG1,k	M-A251	BioLegend	356103
Purified NA/LE mouse antihuman CD28	Mouse IgG1,k	CD28.2	BD Biosciences	555725
APC antihuman CD137 (4-1BB) antibody	Mouse IgG1,k	4B4-1	BioLegend	309810
Alexa Fluor^®^ 647 anti-mouse/rat/human FOXP3 antibody	Mouse IgG1,k	150D	BioLegend	320013
PE/Cyanine7 antihuman CD279 (PD-1) antibody	Mouse IgG1,k	EH12.2H7	BioLegend	329918
Anti-mouse CD3	Rabbit IgG	E4T1B	CellSignaling	78588
Anti-mouse CD4	Rabbit IgG	D7D2Z	CellSignaling	25229
Anti-mouse CD8	Rabbit IgG	D4W2Z	CellSignaling	98941
Anti-mouse FOXP3	Rabbit IgG	D6O8R	CellSignaling	12653
Anti-mouse CD86	Rabbit IgG	E5W6H	CellSignaling	19589
Anti-mouse CD206	Rabbit IgG	E6T5J	CellSignaling	24595
Anti-mouse PD-1	Rabbit IgG	D7D5W	CellSignaling	84651
Anti-mouse 4-1BB	Rabbit IgG	E2J5H	CellSignaling	18798
Anti-mouse CD68	Rabbit IgG	E3O7V	CellSignaling	97778
Human/Mouse/Rat iNOS Antibody	Mouse IgG1	2D2-B2	R&D	MAB9502

### Peripheral blood mononuclear cell preparation

Peripheral blood mononuclear cells (PBMCs) were isolated from human whole blood donations (200–400 mL) from CRS Clinical Research Services Berlin (Berlin, Germany) by density gradient centrifugation.

### CD8^+^ T cell isolation and culture

CD8^+^ T cells were isolated from PBMCs by magnetic-activated cell sorting (MACS), using the REAlease CD8 Microbead Kit (Miltenyi) according to the manufacturer’s instructions. T cells were cultured in X-VIVO 15 Serum-free Hematopoietic Cell Medium (Lonza) on sterile, growth-enhanced treated polystyrene flat-bottom plates (TPP) at 37 °C in a humidified incubator (5% CO_2_). For activation in single and co-culture, CD8^+^ T cells were cultured on 96-well plates (2 × 10^4^ cells in 100 μL per well) and stimulated with 50 ng/mL recombinant human IL-2 (Invitrogen) and anti-CD2/CD3/CD28-coated microbeads, using the T Cell Activation/Expansion Kit (Miltenyi) according to the manufacturer’s instructions. A cell-to-bead ratio of 2:1 (1 × 10^8^ beads per mL) was used. Every 2 days, fresh medium containing the same supplements was added.

### In vitro differentiation of naïve CD4+ T cells to Tregs

Naïve CD4^+^ T cells were isolated from PBMCs with the MagCellect Human Naïve CD^4+^ T Cell kit (R&D), following to the manufacturer’s protocol. For Treg differentiation, plates were pre-coated with antihuman CD3 antibody (5 µg/mL, clone UCHT1, see Table 1) and cells were cultivated in medium supplemented with 5 ng/mL recombinant human TGFβ1 (TGFβ; Gibco), 50 ng/mL IL-2, and anti-CD28 antibody (1 μg/mL, clone CD28.2, see Table 1). Every 3–4 days, fresh medium containing the same supplements was added.

### Macrophage polarization and culture

For macrophage polarization, THP-1 cells (TIB-202, ATCC) were seeded on 6-well plates at an initial concentration of 5 × 10^5^ cells/mL. Cells were stimulated with 5 ng/mL PMA (Sigma) and incubated (37 °C, 5% CO_2_). After 24 h, fresh medium with 5 ng/mL PMA and compounds at 20, 100 or 500 nM or DMSO was added, and the cells were incubated for additional 24 h. On day 3, fresh medium with compounds was added and the cells were stimulated with 20 ng/mL interferon γ (IFNγ; Sigma) and 20 ng/mL lipopolysaccharide (LPS; Sigma) for M1 polarization and 20 ng/mL IL-4 (Miltenyi) for M2 polarization. Control cells (M0) were stimulated with 5 ng/mL PMA. After 24 h, gene expression was analyzed.

Primary monocytes were isolated from PBMCs with the Pan Monocyte Isolation Kit (Miltenyi) and stimulated for 6 days with 20 ng/ml GMCSF (M1 polarization) or with 20 ng/mL MCSF (M2 polarization). Subsequently, cells were stimulated for 24 h with 20 ng/mL LPS and 20 ng/ml IFNγ (*M*1 polarization) or with 20 ng/mL IL-4 (*M*2 polarization) before treatment with copanlisib (100 nM or 1 µM) or DMSO. After 5 days of treatment, gene expression was analyzed.

### Proliferation studies

T cell proliferation was determined using the CellTrace™ Far Red Cell Proliferation Kit (Invitrogen) according to the manufacturer’s instructions. After 6 or 7 days of culture with the incorporated dye, the fluorescence intensity in the allophycocyanin channel was measured in a FACS Canto II cytometer (Beckton Dickinson).

### Flow cytometry

For flow cytometric analysis of surface markers, cells were stained with LiveDead Fixable Aqua Dead Cell Stain (Invitrogen, diluted 1:1000 in PBS) for 30 min (RT, in the dark). After washing, 100 µL of extracellular antibody master mix containing the suggested volume of each antibody for staining 1 × 10^6^ cells (see Table 1) per sample was added and incubated for 10 min (4 °C, in the dark). Controls were incubated in MACS buffer, with 5 μL of each antibody except one, or with the respective isotype controls. For FOXP3 staining, the cells were fixed by adding 300 μL of Fix/Perm buffer (see Table 2) and incubating for 30 min (4 °C). After two washing steps in 300 μL of Perm buffer (see Table 1), FcR blocking reagent (Miltenyi) was applied (according to manufacturer’s instructions). Antihuman FOXP3 antibody was added at the recommended concentration (see Table 1) in Perm buffer and incubated for 30 min (4 °C). Controls were incubated in Perm buffer or with the respective isotype control. Cells were washed with Perm buffer, resuspended in 100 μL of MACS buffer, and 50 µL was analyzed in a FACS Canto II cytometer. The resulting.fcs files were analyzed with FlowLogic (Inivai).

**Table 2:** Buffers used during T cell subset isolation, flow cytometry and cytokine assays

Buffer		Ingredients	Source	Cat#
Fix/Perm Buffer	3× 1×	Dulbecco’s Phosphate Buffered Saline 4 × FOX3P Fix/Perm Buffer	Gibco BioLegend	14190144 421401
MACS Buffer	19× 1×	autoMACS Rising Solution MACS BSA Stock Solution	Miltenyi Biotec Miltenyi Biotec	130-091-222 130-091-376
MagCellect Buffer	9× 1×	UltraPure Distilled Water MagCellect Plus 10 × Buffer	Invitrogen R&D Systems	10977015 895921
Perm Buffer	9× 1×	Dulbecco’s Phosphate Buffered Saline 10 × FOXP3 Perm Buffer	Gibco BioLegend	14190144 421402
REAlease Bead Release Buffer	49× 1×	MACS Buffer REAlease Release Reagent	In-house Miltenyi Biotec	Not applicable 130-117-036
Stopping Buffer	9× 1×	autoMACS Rising Solution MACS BSA Stock Solution	Miltenyi Biotec Miltenyi Biotec	130-091-222 130-091-376

### Cytokine analysis

Secretion of IL-10 in Treg differentiation culture and intratumoral IFNγ and IL-10 levels were measured with U-PLEX kits (MesoScale) according to the manufacturer’s protocol, using a MESO SECTOR S 600 device (MesoScale).

### RT-PCR of gene expression in polarized macrophages

The mRNA expression in THP-1 cells was analyzed by TaqMan RT-PCR (Applied Biosystems). RNA was extracted using the InviTrap^®^ Spin Universal RNA Mini Kit (Stratech) following the manufacturer’s instructions, eluted in 40 µL sterile DNase/RNase-free water, and the concentration was measured with the NanoQuant Plate at the Infinite M1000 reader (Tecan). Reverse transcription was performed using the SuperScript VILO cDNA Synthesis Kit (ThermoFisher) following the manufacturer’s instructions. TaqMan PCR was performed in 384-well format plates (Clear Optical Reaction Plates, Applied Biosystems) with 10 µL reaction volume using the TaqMan Fast Advanced Master Mix (ThermoFisher) following the manufacturer’s instructions. The reactions were run in QuantStudio 7 Flex RT-PCR cycler (Applied Biosystems), and the results were analyzed using the DataAssist software (Applied Biosystems). The following primers from Applied Biosystems were used: CD163, Hs00174705_m1; CD200R, Hs00793597_m1; CD206 (MRC1), Hs00267207_m1; IL10, Hs00961622_m1; CD86, Hs01567026_m1; HLA-DRA, Hs00219575_m1; TNFα, Hs99999043_m1; IL12B, Hs01011518_m1.

For RT-PCR analysis of polarized primary human monocytes, cells were detached by TrypLE Express, collected in fresh medium after detachment, and washed once in 1 × PBS. The cell pellet was dissolved in 330 µL RLT buffer and solution placed onto a Qiashredder and centrifuged for 5 min at 13.000×*g*. RNA isolation was performed according to manufacturer description by addition of the DNase digestion step. RNA was eluted in 30 µL sterile, DNase- and RNase-free water. RNA concentration was measured with the NanoQuant Plate at the Infinite M1000 reader. cDNA synthesis and qRT-PCR were performed according to manufacturer description with primers from Applied Biosystems.

### RNAseq of gene expression in polarized macrophages

For RNA sequencing of THP-1 cells, total RNA of each sample was extracted using the RNeasy^®^ Mini Kit (Qiagen). Manufacturer’s instructions were followed including one additional step of DNase I treatment with the RNase-Free DNase Set (Qiagen) to eliminate genomic DNA. The TruSeq RNA Sample Preparation Kit v2 (Illumina) was used to generate libraries for RNA sequencing from 400 ng RNA in 50 µL. The quality of the generated libraries was assessed with the Agilent DNA 1000 Chip Kit, and DNA quantity was determined using PerfeCta NGS Library Quantification Kit—Illumina Low/ROX (Quanta Bioscience) as provided by the manufacturer. The libraries were pooled, and the final concentration adjusted to 10 nM using Tris-Cl 10 mM, pH 8.5 with 0.1% Tween 20. The pooled library was denatured with 0.1 M NaOH and diluted to 20 pM with pre-chilled HT1 buffer using the HiSeq SBS Reagent Kit v4 for cBot Clustering. The same was performed for the PhiX library (1%). The sample library was mixed with a PhiX Library and applied to a HiSeq v4 flow cell (Illumina) which was subsequently clustered on a cBot (Illumina) using the HiSeq v4 PE cluster Kit (Illumina). The samples were sequenced in the HiSeq® 2500 system according to standard protocols.

### Animal models

Animal experiments were approved by local authorities and conducted in accordance with animal welfare law and the ethical guidelines of Bayer AG or CrownBio (Beijing, China). Female immunocompetent mice (BALB/c in CT26, C57BL/6N in MC38 studies) were inoculated subcutaneously with tumor cells. Tumor size and body weight were monitored two to three times per week. When the tumors reached the predetermined size, the mice were randomized according to tumor size and therapy was started. Copanlisib was dosed intravenously at 14 mg/kg with a 2on/5off schedule. Anti-PD-1 was dosed either intraperitoneally or intravenously at 10 mg/kg or 200 µg/mouse with a twice weekly (BIW) schedule. Tumor volume (0.5 × length × width^2^) was determined based on twice weekly measurement of tumor size by a caliper (length and width). Treatment responses were evaluated by RECIST criteria.

### Immunohistochemistry

Immunohistochemical (IHC) staining was performed with formaldehyde-fixed, paraffin-embedded tumor samples. For each study, tissue microarrays containing 1.5 mm cores from each tumor were prepared, sectioned (3 µM) with a HM355S microtome (ThermoScientific), and stored o.n. at 37 °C, before de-paraffinization with xylene (Sigma-Aldrich), isopropanol and ethanol (96%, 80%, 70%, and 60% dilutions prepared inhouse), and washing in distilled water. Samples were boiled for 20 min in Target Retrieval Solution pH 9 (Dako) and cooled to RT. Slides were encircled with a PAP pen (Dako) and incubated for 30 min with Real Peroxidase Blocking Reagent (Dako), washed twice with PBS-T (0.05% Tween-20, inhouse) incubated for 10 min in Protein Block (Dako), and for 1 h with the respective primary antibody (Table 1). After two washing steps, the samples were incubated for 30 min in the dark with the appropriate HRP-labeled secondary antibody (Envision Polymer System, Dako) and washed twice. Chromogen reaction was performed for 10 min with DAB Chromogen (diluted in DAB Substrate, both from Dako) and stopped by transfer to distilled water. Nuclei were stained with hematoxylin (Dako) for 30 s, and pH shift was reached by continuous tap water flow for at least 10 min. After dehydration with ethanol (60%, 70%, 80%, and 96%), isopropanol and xylene, the slides were mounted with Entellan (ThermoScientific) and imaged with the Pannoramic Scan II (3D Histech). Quantifications of positive cells were performed with QuPath software (version 0.3.2) [35].

### Statistics

Data were analyzed by Mann–Whitney test (when comparing two groups), ordinary one-way analysis of variance (ANOVA) followed by Šídák's multiple comparisons test (when comparing selected groups in one experiment), or nonparametric Kruskal–Wallis test followed by uncorrected Dunn’s test (when comparing all groups from experiment; this was done for all ex vivo data) using GraphPad Prism 9 (GraphPad Software, San Diego, LA, USA). *p* values below 0.05 were considered statistically significant.

Principal component analysis (PCA) of RNAseq data was performed on log2-transformed expression values from all protein coding genes after adding a pseudocount of 0.125 to the data. The p values from t test versus log2 fold changes after addition of a pseudocount of 0.125 to the data were plotted. Hierarchical clustering based on the expression profile of the top 200 most differentially expressed genes across all 27 samples was also performed.

## Results

### Pan-PI3K inhibition prevents the differentiation of CD4+ T cells to Tregs, while having minor effects on CD8^+^ T cell proliferation and activation

**Fig. 1:**
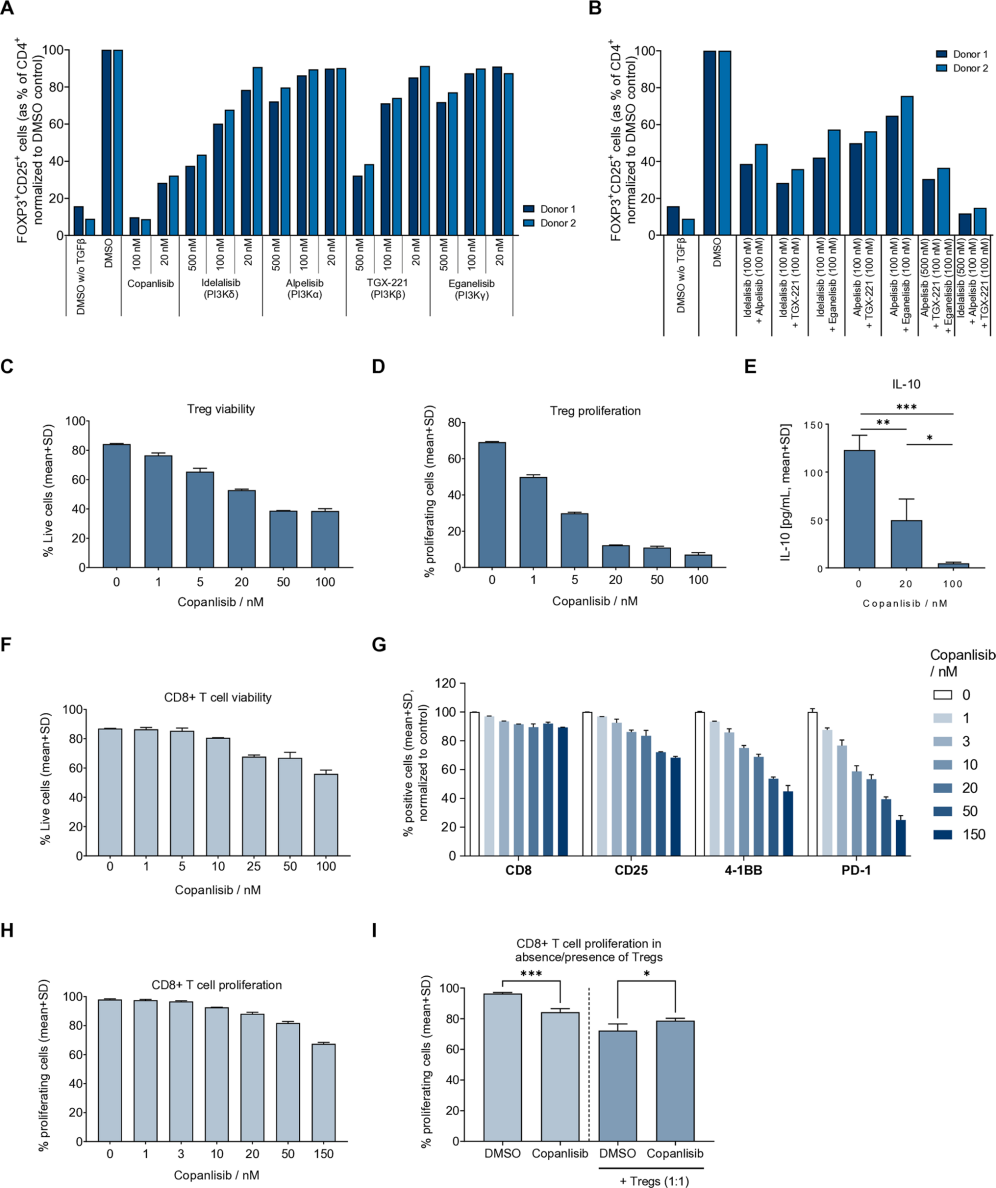
Copanlisib strongly inhibits Treg development, while having minor effects on CD8^+^ T cells in vitro. Naïve CD4^+^ T cells from two healthy donors were differentiated in vitro to Tregs. The expression of FOXP3^+^CD25^+^ was analyzed by flow cytometry after 6 days of treatment with copanlisib (pan-PI3K inhibitor), idelalisib (PI3Kδ inhibitor), alpelisib (PI3Kα inhibitor), TGX-221 (PI3Kβ inhibitor), or eganelisib (PI3Kγ inhibitor) as single agents (**A**) or in combinations (**B**) and shown as % positive cells, normalized to the DMSO control. After 6 days of treatment with copanlisib or DMSO, the viability (**C**) and proliferation (**D**) of cells undergoing in vitro Treg differentiation was assessed by flow cytometry. Data are presented as mean ± SD (*n* = 2). The Secretion of IL-10 in Treg differentiation culture of naïve CD4^+^ T cells was measured after 5 days of treatment with DMSO or copanlisib (**E**). Data are presented as mean + SD (*n* = 3). CD8^+^ T cells were cultured in vitro and treated with copanlisib or DMSO. After 6 days, the viability (**F**) and the expression of T cell activation markers CD25, 4-1BB, and PD-1 (**G**) was assessed by flow cytometry. After 7 days, the proliferation was assessed by flow cytometry (**H**). Data are presented as mean ± SD (*n* = 2). CD8^+^ T cells were cultured with or without Tregs and treated with copanlisib (10 nM) or DMSO. CD8^+^ T cell proliferation was measured by flow cytometry after 6 days (**I**). Data are presented as mean ± SD (*n* = 3). *p* values were calculated by one-way ANOVA and Šídák's multiple comparisons test (*p ≤ 0.05, **p ≤ 0.01, ***p ≤ 0.001)

The conversion of naïve CD4^+^ T cells to Tregs is a central process in tumor immune evasion [16, 17]. To investigate the effects of PI3K inhibition on Treg differentiation in vitro, naïve CD4^+^ T cells were isolated from human whole blood and treated with TGFβ, IL-2, and anti-CD3/CD28 over five days to induce Treg differentiation, followed by the detection of lineage-specific markers forkhead box P3 (FOXP3) and CD25 by flow cytometry [36]. Treatment with the pan-PI3K inhibitor copanlisib inhibited the upregulation of Treg markers in vitro more efficaciously than the isoform-selective PI3K inhibitors idelalisib (CAL-101, PI3Kδ), alpelisib (BYL-719, PI3Kα), TGX-221 (PI3Kβ), and eganelisib (IPI-549, PI3Kγ), when administered alone (Fig. 1A, see Suppl. Fig. S1A for representative flow cytometry histograms). Combination of idelalisib, alpelisib, and TGX-221, on the other hand, was effective in inhibiting FOXP3 and CD25 expression, leading to the conclusion that inhibiting multiple forms of PI3K is more efficient in suppressing Treg differentiation (Fig. 1B).

Next, the viability of cells undergoing Treg differentiation was assessed by flow cytometry. Copanlisib dose-dependently reduced Treg viability to less than 40% at concentrations of 50 nM or higher (Fig. 1C). Furthermore, we analyzed Treg proliferation using the CellTrace detection system, where the proliferation of cells is indicated by the reduction of fluorescence intensity of an incorporated dye. Copanlisib strongly reduced the percentage of proliferating cells in Treg differentiation culture to less than 20% at concentrations of 20 nM or higher (Fig. 1D).

In order to analyze the effect of pan-PI3K inhibition on the functionality of induced Tregs, we measured the secretion of the Treg effector cytokine IL-10 in supernatants of Treg differentiation culture [36]. Copanlisib dose-dependently reduced the secretion of IL-10 (Fig. 1E), indicating that pan-PI3K inhibition does not only impair the differentiation, viability, and proliferation of Tregs, but also affects their functionality.

Next, the effects of copanlisib on cytotoxic T cells were investigated in vitro. CD8^+^ T cells were isolated from human whole blood, stimulated with anti-CD2/CD3/CD28, and treated with copanlisib or dimethyl sulfoxide (DMSO), and the viability was determined by flow cytometry. In contrast to Tregs, the viability of CD8^+^ T cells was reduced only to approx. 60% at the highest concentration of 100 nM copanlisib (Fig. 1F).

In order to elucidate the role of PI3K in CD8^+^ T cell activation, selected surface markers were analyzed by flow cytometry. All analyzed markers were reduced by copanlisib treatment, however in a different susceptibility: The expression of the lineage marker CD8 and the activation marker CD25 was slightly reduced to approx. 90 and 70%, respectively, when comparing the highest concentration to the control cells (Fig. 1G). The activation markers 4-1BB and PD-1 were more strongly affected and reduced to below 50 and 30%, respectively.

Finally, the effect of pan-PI3K inhibition on CD8^+^ T cell proliferation was determined using the CellTrace system. Copanlisib inhibited the proliferation of freshly isolated CD8^+^ T cells only to 70% at the highest concentration of 150 nM (Fig. 1H). Notably, this was a much weaker effect than the inhibition of Treg proliferation (Fig. 1D). To assess if this effect of copanlisib on CD8^+^ T cell proliferation could diminish its advantageous anti-Treg effect, we conducted co-culture experiments with in vitro-induced Tregs and autologous CD8^+^ T cells that were stained with the CellTrace detection system. Again, copanlisib reduced the proliferation of CD8^+^ T cells when the cells were cultured without Tregs; however, to our surprise, when CD8^+^ T cells were co-cultured in 1:1 ratio with in vitro-induced Tregs, the effect of PI3K inhibition was reversed, leading to a small but significant increase of CD8^+^ T cell proliferation (Fig. 1I). This underlines that copanlisib has functional effects on Tregs, which appear to be stronger than the inhibition of CD8^+^ T cell proliferation, providing a net positive effect in Treg-dominated environments.

### Pan-PI3K inhibition by copanlisib promotes M1 macrophage polarization

**Fig. 2:**
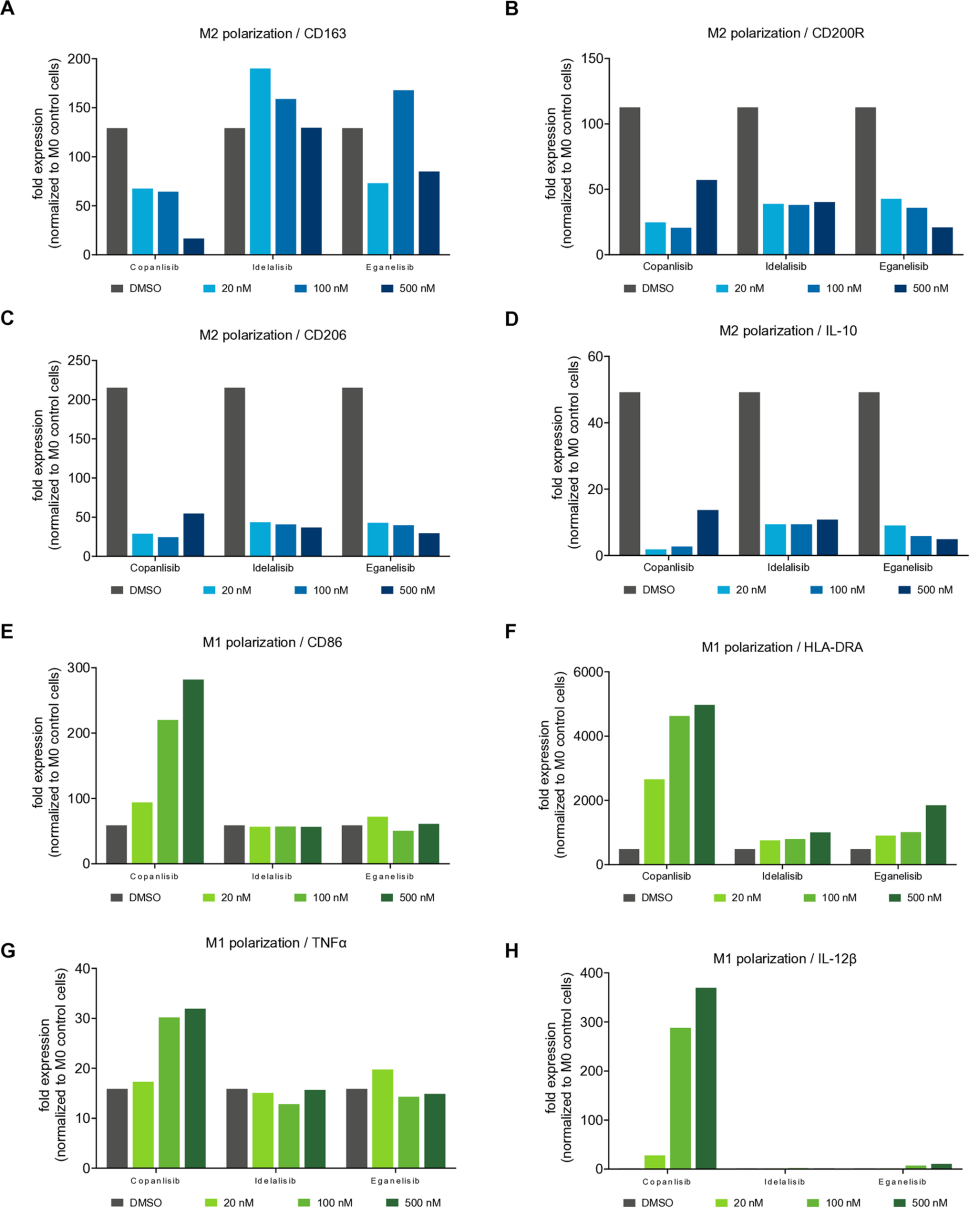
Copanlisib reduces *M*2 macrophage gene expression and promotes *M*1 marker upregulation in THP-1 cells. mRNA expression of the *M*2 macrophage marker genes CD163 (**A**), CD200R (**B**), CD206 (**C**), and IL-10 (**D**), or the *M*1 markers CD86 (**E**), HLA-DRA (**F**), TNFα (**G**), and IL-12β (**H**), following treatments with copanlisib, idelalisib, and eganelisib in THP-1 acute monocytic leukemia cells cultured in *M*1 or *M*2 macrophage polarization medium. mRNA levels were quantified by qPCR and normalized to unpolarized (*M*0) THP-1 cells

M1 and M2 TAM subsets play opposite roles in anti-tumor immunity. The abundance of M2 TAMs is an important aspect of tumor immune evasion and therefore cancer immunotherapies aim to shift macrophage polarization more toward the pro-inflammatory M1 subset. To analyze the effect of PI3K inhibition on macrophage polarization, we used the THP-1 acute monocytic leukemia cell line as in vitro model. THP-1 cells were polarized toward M1 macrophages (with IFNγ and LPS) or M2 macrophages (with IL-4), while control cells (M0) were stimulated with PMA. During polarization, cells were treated with DMSO or with copanlisib, idelalisib, or eganelisib over a broad concentration range from 20 to 500 nM, covering the clinically relevant dose levels. By qPCR, we measured the RNA levels of selected *M*1 and *M*2 markers. Compared to both isoform-selective inhibitors, copanlisib more effectively reduced the expression of the M2 marker gene CD163 (Fig. 2A), whereas all compounds were found to similarly inhibit the expression of the M2 markers CD200R, CD206, and IL-10 (Fig. 2B–D). The analysis of M1 marker genes, on the other hand, showed a differential outcome for the tested PI3K inhibitors. Importantly, only copanlisib induced a dose-dependent upregulation of the M1 markers CD86, HLA-DRA, TNFα, and IL-12β, while the two isoform-selective PI3K inhibitors did not (Fig. 2E–H). These data suggest that pan-PI3K inhibition promotes the polarization of M1 macrophages while at the same time preventing *M*2 polarization.

**Fig. 3:**
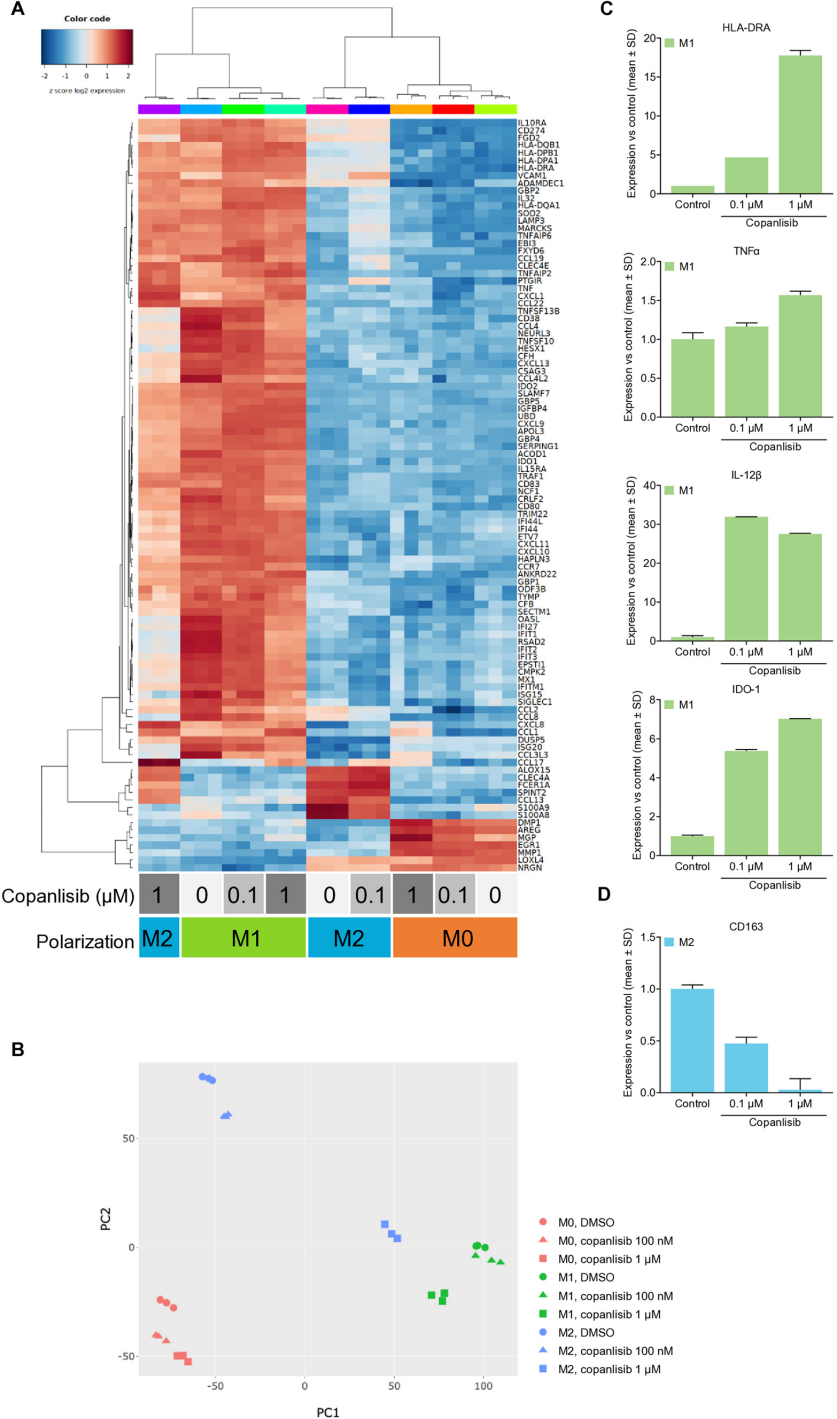
Pan-PI3K inhibition by copanlisib inhibits *M*2 polarization of THP-1 cells and primary human monocytes. THP-1 acute monocytic leukemia cells were polarized toward *M*1 and *M*2 macrophages or cultured as control cells (M0) while being treated with copanlisib (100 nM or 1 µM) or DMSO. Gene expression was analyzed by RNAseq and visualized by hierarchical clustering (**A**) and principal component analysis of 20.000 genes (**B**). Human primary monocytes were polarized toward *M*1 macrophages (**C**) or *M*2 macrophages (D) before treatment with copanlisib and analysis for the expression of selected *M*1 marker genes (**C**) or the *M*2 marker CD163 (**D**). Data were obtained by qPCR and are presented as mean + SD (*n* = 2–3) relative to the DMSO control

The effect of copanlisib on *M*1 and *M*2 polarization was analyzed in more detail by RNAseq. As expected, THP-1 cells upregulated *M*1 marker genes under conditions of *M*1 polarization, while *M*2-polarized THP-1 cells expressed *M*2 marker genes (Fig. 3A). However, treatment with copanlisib inhibited *M*2 polarization, resulting in THP-1 cells that expressed *M*1 genes despite being cultured under *M*2 polarization conditions. Dendrogram assignment of the RNAseq data indicated that the transcriptome of *M*2-polarized THP-1 cells treated with high doses of copanlisib resembled more closely the expression profile of *M*1-polarized THP-1 cells, rather than *M*2-polarized control cells treated with DMSO. This was confirmed by principal component analysis of all 20,000 genes that were analyzed (Fig. 3B). Moreover, copanlisib upregulated a comprehensive set of pro-inflammatory cytokines and IFNγ-induced genes, promoted the expression of *M*1-specific genes such as CXCL10, and strongly downregulated classical *M*2 markers such as CD163 (Suppl. Fig S1. B–D). In contrast, copanlisib treatment did not generally modify the transcription patterns of *M*1- or *M*0-polarized THP-1 cells.

Finally, we analyzed human primary monocytes that were polarized into *M*1 and *M*2 macrophages prior to treatment with copanlisib. In this experiment, copanlisib treatment further increased the expression of *M*1 markers HLA-DRA, TNFα, IL-12β, and IDO-1 in *M*1-polarized macrophages (Fig. 3C). However, it downregulated the expression of the *M*2 marker CD163 in *M*2-polarized cells (Fig. 3D). The tested in vitro doses of copanlisib are physiologically relevant, as these concentrations are reached in vivo in preclinical tumor models and are also in a clinically relevant dose range [37, 38]. This underlines that copanlisib treatment does not only inhibit in vitro *M*2 polarization but can also repolarize *M*2 macrophages toward the *M*1 phenotype, further strengthening the hypothesis that pan-PI3K inhibition can modulate the TME to become more prone to anti-tumor immune responses.

### Copanlisib induces immunomodulation and anti-tumor activity in the ICI-insensitive tumor model MC38

To study the anti-tumor activity and the immunomodulatory effects of copanlisib in vivo, the pan-PI3K inhibitor was applied alone and in combination with the ICI anti-PD-1 antibody in two syngeneic mouse models. As established in our previous studies, copanlisib was dosed intermittently (2on/5off), while anti-PD-1 was given twice per week. Tumor models were selected based on their immune infiltration profile and their response to ICI in vivo, as well as the direct effects of PI3K inhibitors on their in vitro proliferation. Copanlisib inhibited the proliferation of MC38 cells in vitro with an IC50 of approx. 500 nM (Suppl. Fig S1E). In contrast, it had only a weak effect on the in vitro proliferation of CT26 cells (IC50 of approx. 38 µM), supporting previous findings that CT26 cells are resistant to PI3K inhibition in vitro and in vivo in immunodeficient nude mice [29].

**Fig. 4:**
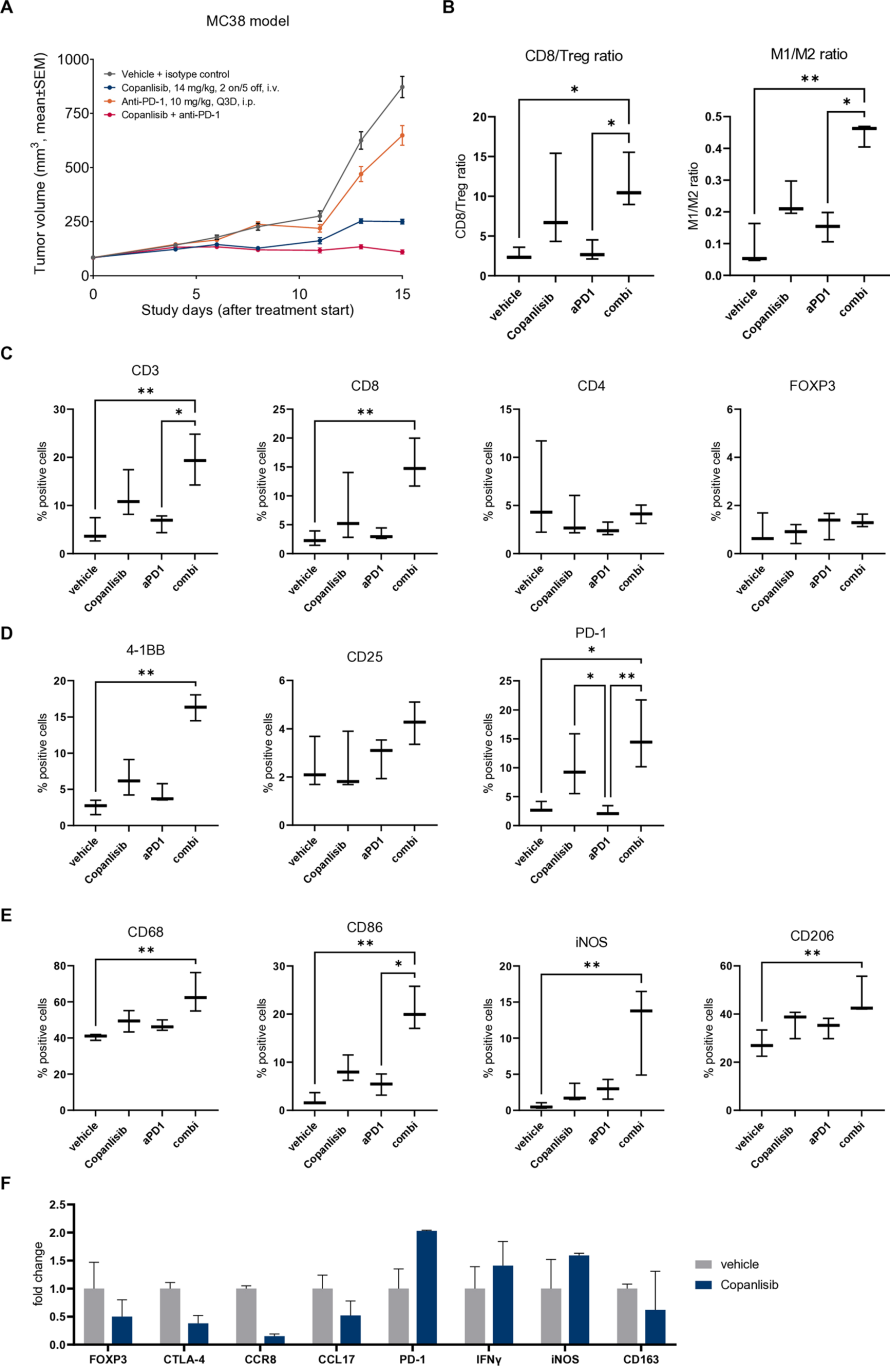
Copanlisib inhibits tumor growth and immune evasion in combination with anti-PD-1 in ICI-insensitive MC38 model. Female immunocompetent C57BL/6N mice were inoculated with MC38 tumors and treated with copanlisib and/or anti-PD-1 at the indicated doses and dosing schedules (**A**). Tumor growth data are presented as mean ± SEM (*n* = 10–16). Biomarker samples were collected on day 13 and analyzed by IHC for immune cell infiltrates. T cell markers (**C**), T cell activation markers (**D**), and macrophage markers (**E**) were quantified and presented as percentage of all cells (*n* = 3). From CD8^+^ and FOXP3^+^ cell percentages, the CD8/Treg ratio was calculated (**B**, left) and from CD86^+^ and CD206^+^ cell percentages, the *M*1/*M*2 macrophage ratio was calculated (B, right). *p*-values were calculated by Kruskal–Wallis and uncorrected Dunn’s test for multiple comparisons between all groups (*p ≤ 0.05, **p ≤ 0.01, ***p ≤ 0.001). The expression of selected genes in MC38 tumors from satellite animals treated with vehicle or copanlisib was analyzed by qPCR (**F**). Data are presented as fold change compared to the vehicle group (*n* = 2)

First, we analyzed the immunomodulatory effects of copanlisib in the MC38 colorectal cancer model that shows moderate to weak responses to ICI [32]. MC38 tumors exhibit high numbers of macrophages, especially of immunosuppressive, tumor promoting *M*2 TAMs. While anti-PD-1 monotherapy showed no significant anti-tumor activity compared to the control group, the efficacy of copanlisib monotherapy was significant (Fig. 4A). Importantly, when copanlisib was combined with anti-PD-1, the efficacy was further enhanced. IHC analysis of tumor infiltrates revealed that combination treatment significantly increased the presence of CD3^+^ and CD8^+^ T cells, while not significantly altering the levels of CD4^+^ T cells or FOXP3^+^ Tregs (Fig. 4C, see Suppl. Fig. 2A for representative IHC staining of vehicle and combination group). Furthermore, the abundance of activation markers 4-1BB and PD-1 was significantly elevated and a trend to increased CD25 expression was seen (Fig. 4D). In addition, combination of copanlisib and anti-PD-1 provoked higher levels of CD68^+^ macrophages and strongly increased the presence of *M*1 markers CD86 and iNOS, while the *M*2 marker CD206 was only slightly changed (Fig. 4E). This resulted in a significant increase of both the CD8/Treg and the *M*1/*M*2 ratio in combination therapy, when compared to the control and anti-PD-1 groups (Fig. 4B).

Copanlisib monotherapy showed similar but insignificant trends in IHC analysis. To further address its immunomodulatory effects, tumor samples from the copanlisib monotherapy group were analyzed by qPCR and compared to the vehicle group. Here, a trend toward the reduction of Treg markers FOXP3 and CTLA-4 was detected (Fig. 4F). Especially CCR8, which is a marker for activated Tregs, was strongly downregulated by copanlisib, and the Treg-recruiting chemokine CCL17 was slightly reduced [36]. The T cell activation marker PD-1 was strongly increased, confirming our IHC data (Fig. 4D). Also, the pro-inflammatory cytokine IFNγ was slightly elevated on RNA level (Fig. 4F). These results were supported by another independent MC38 study, where copanlisib monotherapy and combination treatment with anti-PD-1 led to significant increases of intratumoral IFNγ and a tendency to reduced IL-10 secretion (Suppl. Fig. 2B). Finally, trends toward an increase of the M1 marker iNOS and a reduction of the *M*2 marker CD163 were found on mRNA level (Fig. 4F), standing in line with our findings from in vitro experiments on macrophage polarization (Figs. 2 and 3A–D). Taken together, these data underline that pan-PI3K inhibition with copanlisib ameliorates anti-tumor immunity and enhances sensitivity to ICI in the syngeneic mouse tumor model MC38.

### Copanlisib shows in vivo activity in PI3K inhibition-resistant CT26 tumors and causes complete remission and anti-tumor memory formation in combination with ICI

**Fig. 5:**
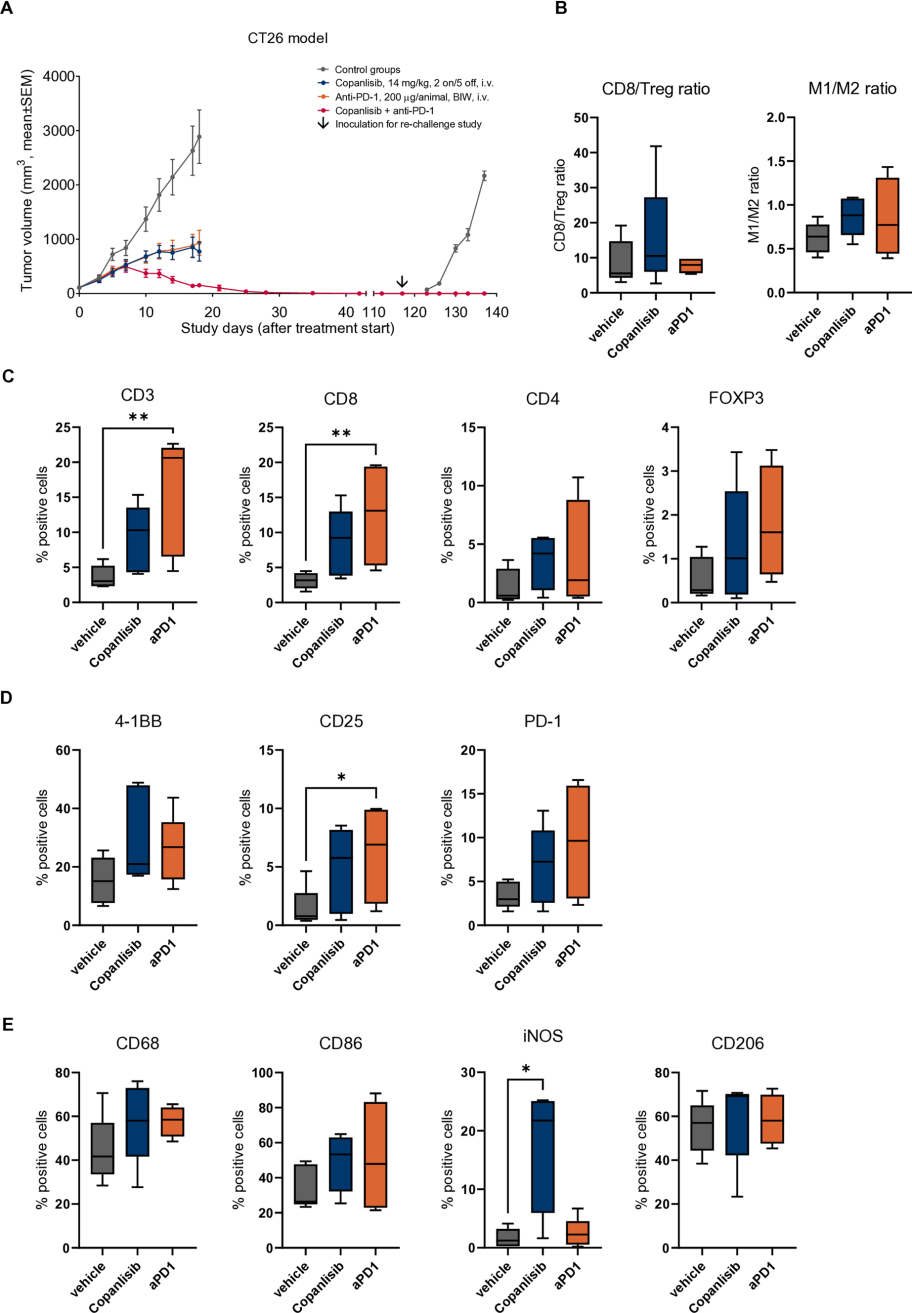
Copanlisib shows in vivo efficacy in PI3K inhibitor-resistant CT26 model, causes complete remission and anti-tumor memory with anti-PD-1. Female immunocompetent BALB/c mice were inoculated with CT26 tumors and treated with copanlisib and/or anti-PD-1 at the indicated doses and dosing schedules (**A**). Tumor growth data are presented as mean ± SEM (*n* = 5–7 for efficacy study; *n* = 3 for re-challenge study). Biomarker samples were collected on day 18 and analyzed by IHC for immune cell infiltrates. T cell markers (**C**), T cell activation markers (**D**), and macrophage markers (E) were quantified and presented as percentage of all cells (*n* = 4–5). From CD8^+^ and FOXP3^+^ cell percentages, the CD8/Treg ratio was calculated (**B**, left) and from CD86^+^ and CD206^+^ cell percentages, the *M*1/*M*2 macrophage ratio was calculated (**B**, right). *p*-values were calculated by Kruskal–Wallis and uncorrected Dunn’s test for multiple comparisons between all groups (*p ≤ 0.05, **p ≤ 0.01, ***p ≤ 0.001)

Next, we analyzed the immunomodulatory capabilities of copanlisib in the murine colon cancer model CT26 which is characterized by a high proportion of infiltrating macrophages and NK cells [39]. The CT26 model is ICI-sensitive, which was confirmed by reduced tumor growth under anti-PD-1 treatment (Fig. 5A). Although copanlisib had only weak effects on CT26 cell proliferation in vitro (Suppl. Fig. S1E), it strongly reduced CT26 tumor growth in vivo (Fig. 5A). Combination of copanlisib with anti-PD-1 was more efficacious than both monotherapies and led to complete tumor regression after 4 weeks of treatment. Mice from the combination group survived without tumor relapse after the end of treatment and were used for a re-challenge study beginning 3 months after the final treatment. In contrast to control mice, no tumor growth was observed in mice that had previously received the combination of copanlisib and anti-PD-1 (Fig. 5A), suggesting that tumor-specific memory cells have been generated.

Due to complete remission in the combination group, no tumor samples were available for biomarker analysis. Samples from the control and monotherapy groups were analyzed for immune cell infiltration by IHC. Here, significant increases of CD3^+^ and CD8^+^ T cells were found in the anti-PD-1 group (Fig. 5C). Under copanlisib treatment, a trend to elevated levels of CD3^+^, CD8^+^, and CD4^+^ T cells and FOXP3^+^ Tregs was observed. Similarly, a trend to increased abundance of the activation markers 4-1BB, CD25, and PD-1 was found in the copanlisib group, while anti-PD-1 treatment significantly increased the percentage of CD25^+^ cells (Fig. 5D). Infiltration of CD68^+^ macrophages tended to increase in both monotherapies, accompanied by a trend to higher numbers of CD86^+^ cells (Fig. 5E). In the copanlisib group, the M1 marker iNOS was significantly increased, while the M2 marker CD206 was not significantly affected. These IHC analyses resulted in slightly increased CD8/Treg and M1/M2 ratios in the copanlisib group, compared to the control and the anti-PD-1 monotherapy group (Fig. 5B). This tendency was further confirmed by flow cytometry data from another CT26 biomarker study, in which copanlisib monotherapy caused a significantly increased CD8/Treg ratio already after one cycle of treatment due to a strong depletion of intratumoral Tregs (Suppl. Fig. 3). Furthermore, the expression of the activation marker 4-1BB on the remaining intratumoral Treg population was significantly reduced by copanlisib.

In conclusion, we demonstrated that copanlisib has an anti-tumor activity in the CT26 model even though the same cell line is PI3K inhibition-resistant in vitro. This in vivo efficacy is likely mediated via immunomodulatory effects of copanlisib that also lead to improved efficacy of ICI, resulting in complete tumor remission and the development of an anti-tumor immune memory.

## Discussion

The PI3K pathway is an important target in cancer therapy, not only because its activation can increase the proliferation and survival of cancer cells, but also because it creates an immunosuppressive TME, resulting in resistance to ICIs [4, 20, 22, 40–42]. PI3K inhibitors therefore represent an important therapeutic approach, particularly in the case of ICI-resistant tumors. In this study, we have shown the anti-tumor efficacy of the pan-PI3K inhibitor copanlisib as a single agent in two murine syngeneic tumor models. In the ICI-insensitive MC38 model, the limited response to anti-PD-1 treatment was overcome by combination with copanlisib. In the ICI-susceptible CT26 model, the combination of copanlisib and anti-PD-1 led to stronger in vivo efficacy, resulting in complete tumor remission and the development of an immunological memory toward cancer cells, as none of the animals were found to develop tumors upon re-challenge with the same cell line. This stands in line with previous findings on combination therapy of isoform-selective PI3K inhibitors with anti-PD-1 [31]. Since CT26 cells are resistant to PI3K inhibition in vitro and in vivo (when using immunodeficient mice), the observed in vivo anti-tumor efficacy in immunocompetent mice can be clearly linked to immunomodulatory effects, as previously reported for other PI3K inhibitors [29].

The TME contains several types of immune cells, including T cells, myeloid cells, natural killer cells and B cells [43]. Within these cell types, various subsets have been characterized which exert either pro-inflammatory anti-tumor effects or immunosuppressive, tumor-promoting functions. The relative abundance of these subsets and their modulation by PI3K inhibitors may have substantial influence on therapeutic success. In this study, we focused on the subsets of cytotoxic T cells and Tregs, as well as M1 and M2 macrophages, as main modulators of the balance between suppressive and stimulatory functions and studied the effects of copanlisib on these immune cell types in vitro and in vivo.

Tregs suppress anti-tumor immunity and are associated with a poor prognosis in patients with various types of cancer. Our in vitro studies demonstrated that pan-PI3K inhibition with copanlisib inhibits the differentiation of naïve CD4^+^ T cells into Tregs and impairs the viability and proliferation of in vitro-induced Tregs as well as their function to secrete the immunosuppressive cytokine IL-10. In contrast, several isoform-selective PI3K inhibitors could not suppress in vitro Treg differentiation to a similar extent as copanlisib. Only the combination of three isoform-selective inhibitors was nearly as effective; therefore it can be concluded that pan-PI3K inhibition is required to fully inhibit Treg differentiation.

The infiltration of cytotoxic CD8^+^ T cells into tumors has been demonstrated to be a significant predictor for the efficacy of ICIs [3–6], and thus, any therapy intended to be used concomitantly with ICIs should not impair CD8^+^ T cell abundance and functionality. Although copanlisib reduced the viability and proliferation of CD8^+^ T cells in vitro, this effect was less prominent than the inhibition of Treg proliferation. These findings stand in line with previous publications showing a stronger effect of PI3K inhibitors on Tregs than on CD8^+^ T cells [44] and were underlined by our co-culture experiments of CD8^+^ T cells with in vitro-induced Tregs. Here, CD8^+^ T cell proliferation was enhanced by copanlisib treatment, suggesting that pan-PI3K inhibition primarily impairs the functionality of Tregs, which then results in a net increase of CD8^+^ T cell proliferation.

The T cell-modulatory effects of copanlisib were confirmed by our in vivo studies: In both murine syngeneic models, trends toward increased infiltration of overall CD3^+^ T cells and cytotoxic CD8^+^ T cells were shown upon copanlisib treatment. In combination with anti-PD-1, these effects were even stronger, resulting in significantly increased cytotoxic T cell infiltration and, since the Treg levels were not significantly altered, in an elevated CD8/Treg ratio in the MC38 model. Due to complete tumor remission in the CT26 model, no samples from the combination group could be analyzed; however, the anti-PD-1 monotherapy caused significantly increased intratumoral CD3 and CD8 levels, and the copanlisib group showed the same trend. Therefore, a similar or even stronger effect on T cell infiltration could be assumed for the combination group and may be partially responsible for the observed strong anti-tumor efficacy. Notably, we have observed in several studies that the immunomodulatory effects of copanlisib are highly tumor-specific, which may lead to its favorable tolerability, bringing along the drawback that no other tissues can be used as surrogate biomarker (data not shown). In a separate CT26 biomarker study, monotherapy with copanlisib caused a significantly elevated CD8/Treg ratio due to depletion of intratumoral Tregs, while the CD8^+^ T cell level was slightly increased. On the remaining intratumoral Tregs, the expression of the activation marker 4-1BB was significantly reduced by copanlisib, indicating that activated Tregs were primarily affected. The analysis of intratumoral RNA levels in the MC38 model confirmed the anti-Treg effect of copanlisib, especially regarding CCR8^+^ activated Tregs. This stands in line with our in vitro observations and indicates a superior sensitivity of qPCR compared to IHC.

Importantly, when looking at overall immune cell infiltrates, copanlisib treatment increased the intratumoral presence of 4-1BB^+^, CD25^+^, and PD-1^+^ cells alone and, more strongly, in combination with anti-PD-1. This indicates that the infiltrating cytotoxic T cells are largely in an activated state and may seem to contradict our in vitro data, where copanlisib slightly reduced the expression of those markers. However, it should be considered that the conditions in the TME are more complex and that the temporal regulation of surface marker expression on tumor infiltrating T cells is influenced by a variety of factors (such as presence of other cell types like macrophages, see below) that cannot be fully reflected in vitro. Previous publications on other PI3K inhibitors have shown similar increases of intratumoral T cell activation marker levels [29]. The increased T cell activation could be due to the pre-dominant anti-Treg effect of copanlisib, which is stronger than its suppression of CD8^+^ T cells. This is further supported by intratumoral cytokine measurements showing increased levels of IFNγ, while IL-10 tended to be reduced. In conclusion, in addition to its direct anti-tumor effects, copanlisib modulates the intratumoral T cell compartment in a favorable way, inhibiting Tregs and promoting activated cytotoxic CD8^+^ T cells, which exert a strong anti-tumor immunity.

In addition to T cells, macrophages play an important role in anti-tumor immunity [45, 46]. TAMs have two polarization states: While *M*1 macrophages produce pro-inflammatory cytokines, such as IL-12β, and TNFα, and are considered as immunostimulatory and thus anti-tumorigenic [47], *M*2 TAMs produce anti-inflammatory cytokines, such as IL-10, IL-13, and TGFβ, that promote an immunosuppressive environment and thus tumor growth [48]. The polarization of macrophages is therefore a crucial factor for immune responses in solid tumors. In this study, we demonstrated that copanlisib simultaneously promotes *M*1 polarization and inhibits *M*2 polarization in vitro in THP-1 cells and polarized primary monocytes. Importantly, copanlisib had superior effects compared to isoform-selective PI3K inhibitors, indicating that PI3K isoforms may be involved differently in macrophage polarization and that only pan-PI3K inhibition can effectively promote the *M*1 phenotype.

In both syngeneic mouse tumor models, the infiltration of CD68^+^ macrophages was increased by copanlisib alone and in combination with anti-PD-1. This was accompanied by elevated levels of the *M*1 macrophage markers CD86 and iNOS but also to a certain extent of the *M*2 marker CD206 in both mouse models [49]. Although both populations were increased, the quantifications resulted in a significantly higher *M*1/*M*2 ratio in the MC38 model upon combination therapy. In the same study, a similar trend was shown for copanlisib monotherapy by qPCR and IHC. In the CT26 model, the number of iNOS^+^ *M*1 macrophages was significantly elevated upon copanlisib treatment. As it was shown before, the *M*1 phenotype of TAMs may boost memory T cell infiltration [47], which provides an explanation for the anti-tumor memory observed in the re-challenge study of the combination group. Concluding, it was shown that copanlisib shifts the balance of TAMs from the *M*2 toward the *M*1 state, thereby promoting direct anti-tumor immunity and reducing the immunosuppressive factors, which might also improve the previously described beneficial effects of copanlisib on T cells.

## Conclusions

Intermittent pan-PI3K inhibition with copanlisib provides an effective strategy to influence the microenvironment of solid tumors through the increased infiltration of T cells and macrophages, the improvement of both CD8/Treg and *M*1/*M*2 ratios, and the increased activation of T cells and production of pro-inflammatory cytokines. Thereby, it overcomes ICI insensitivity and shows enhanced efficacy in combination with anti-PD-1 treatment. This supports further investigation of copanlisib in combination with ICIs in ICI-resistant tumors.\

### Supplementary Information

Below is the link to the electronic supplementary material.

Supplementary file1 (PDF 558 KB)

## Data Availability

Data are available from the authors upon reasonable request and with permission of Bayer AG.

## References

[1] Sharma P, Allison JP (2015). Immune checkpoint targeting in cancer therapy: toward combination strategies with curative potential. Cell.

[2] Sharma P, Allison JP (2020). Dissecting the mechanisms of immune checkpoint therapy. Nat Rev Immunol.

[3] Daud AI, Loo K, Pauli ML, Sanchez-Rodriguez R, Sandoval PM, Taravati K, Tsai K, Nosrati A, Nardo L, Alvarado MD (2016). Tumor immune profiling predicts response to anti-PD-1 therapy in human melanoma. J Clin Invest.

[4] Peng W, Chen JQ, Liu C, Malu S, Creasy C, Tetzlaff MT, Xu C, McKenzie JA, Zhang C, Liang X (2016). Loss of PTEN promotes resistance to T cell-mediated immunotherapy. Cancer Discov.

[5] Spranger S, Bao R, Gajewski TF (2015). Melanoma-intrinsic beta-catenin signalling prevents anti-tumour immunity. Nature.

[6] Tumeh PC, Harview CL, Yearley JH, Shintaku IP, Taylor EJ, Robert L, Chmielowski B, Spasic M, Henry G, Ciobanu V (2014). PD-1 blockade induces responses by inhibiting adaptive immune resistance. Nature.

[7] Jansen CS, Prokhnevska N, Kissick HT (2019). The requirement for immune infiltration and organization in the tumor microenvironment for successful immunotherapy in prostate cancer. Urol Oncol.

[8] Zou W, Wolchok JD, Chen L (2016). PD-L1 (B7–H1) and PD-1 pathway blockade for cancer therapy: mechanisms, response biomarkers, and combinations. Sci Transl Med.

[9] Bonaventura P, Shekarian T, Alcazer V, Valladeau-Guilemond J, Valsesia-Wittmann S, Amigorena S, Caux C, Depil S (2019). Cold tumors: a therapeutic challenge for immunotherapy. Front Immunol.

[10] Galluzzi L, Chan TA, Kroemer G, Wolchok JD, Lopez-Soto A (2018). The hallmarks of successful anticancer immunotherapy. Sci Transl Med.

[11] Joyce JA, Fearon DT (2015). T cell exclusion, immune privilege, and the tumor microenvironment. Science.

[12] Scheffold A, Hanna BS, Roeßner P, Demerdash Y, Jebaraj BMC, Lichter P, Stilgenbauer S, Seiffert M (2017). PI3K-δ inhibition influences T-cell populations and anti-tumoral immune function in preclinical models. Blood.

[13] Ali K, Soond DR, Pineiro R, Hagemann T, Pearce W, Lim EL, Bouabe H, Scudamore CL, Hancox T, Maecker H (2014). Inactivation of PI(3)K p110delta breaks regulatory T-cell-mediated immune tolerance to cancer. Nature.

[14] Parker KH, Beury DW, Ostrand-Rosenberg S (2015). Myeloid-derived suppressor cells: critical cells driving immune suppression in the tumor microenvironment. Adv Cancer Res.

[15] Chen Y, Song Y, Du W, Gong L, Chang H, Zou Z (2019). Tumor-associated macrophages: an accomplice in solid tumor progression. J Biomed Sci.

[16] Waniczek D, Lorenc Z, Snietura M, Wesecki M, Kopec A, Muc-Wierzgon M (2017). Tumor-associated macrophages and regulatory T cells infiltration and the clinical outcome in colorectal cancer. Arch Immunol Ther Exp (Warsz).

[17] Paluskievicz CM, Cao X, Abdi R, Zheng P, Liu Y, Bromberg JS (2019). T regulatory cells and priming the suppressive tumor microenvironment. Front Immunol.

[18] Cheng J, Huang Y, Zhang X, Yu Y, Wu S, Jiao J, Tran L, Zhang W, Liu R, Zhang L (2020). TRIM21 and PHLDA3 negatively regulate the crosstalk between the PI3K/AKT pathway and PPP metabolism. Nat Commun.

[19] Okkenhaug K, Graupera M, Vanhaesebroeck B (2016). Targeting PI3K in cancer: impact on tumor cells, their protective stroma, angiogenesis, and immunotherapy. Cancer Discov.

[20] Sivaram N, McLaughlin PA, Han HV, Petrenko O, Jiang YP, Ballou LM, Pham K, Liu C, van der Velden AW, Lin RZ (2019). Tumor-intrinsic PIK3CA represses tumor immunogenecity in a model of pancreatic cancer. J Clin Invest.

[21] Thorpe LM, Yuzugullu H, Zhao JJ (2015). PI3K in cancer: divergent roles of isoforms, modes of activation and therapeutic targeting. Nat Rev Cancer.

[22] Wu S, Zhang Q, Zhang F, Meng F, Liu S, Zhou R, Wu Q, Li X, Shen L, Huang J (2019). HER2 recruits AKT1 to disrupt STING signalling and suppress antiviral defence and antitumour immunity. Nat Cell Biol.

[23] Bilanges B, Posor Y, Vanhaesebroeck B (2019). PI3K isoforms in cell signalling and vesicle trafficking. Nat Rev Mol Cell Biol.

[24] Jia S, Liu Z, Zhang S, Liu P, Zhang L, Lee SH, Zhang J, Signoretti S, Loda M, Roberts TM (2008). Essential roles of PI(3)K-p110beta in cell growth, metabolism and tumorigenesis. Nature.

[25] Wang X, Ding J, Meng LH (2015). PI3K isoform-selective inhibitors: next-generation targeted cancer therapies. Acta Pharmacol Sin.

[26] Soond DR, Bjørgo E, Moltu K, Dale VQ, Patton DT, Torgersen KM, Galleway F, Twomey B, Clark J, Gaston JSH (2010). PI3K p110δ regulates T-cell cytokine production during primary and secondary immune responses in mice and humans. Blood.

[27] Lim EL, Cugliandolo FM, Rosner DR, Gyori D, Roychoudhuri R, Okkenhaug K (2018). Phosphoinositide 3-kinase delta inhibition promotes antitumor responses but antagonizes checkpoint inhibitors. JCI Insight.

[28] Koblish HK, Wang L-C, Hansbury M, Zhang Y, Yang G, Burn T, Waeltz P, Rupar M, Yue E, Douty B (2015). Abstract C103: the combination of PI3kδ-selective inhibition and immunomodulation shows efficacy in solid tumor models. Mol Cancer Ther.

[29] Carnevalli LS, Sinclair C, Taylor MA, Gutierrez PM, Langdon S, Coenen-Stass AML, Mooney L, Hughes A, Jarvis L, Staniszewska A (2018). PI3Kalpha/delta inhibition promotes anti-tumor immunity through direct enhancement of effector CD8(+) T-cell activity. J Immunother Cancer.

[30] De Henau O, Rausch M, Winkler D, Campesato LF, Liu C, Cymerman DH, Budhu S, Ghosh A, Pink M, Tchaicha J (2016). Overcoming resistance to checkpoint blockade therapy by targeting PI3Kgamma in myeloid cells. Nature.

[31] Kaneda MM, Cappello P, Nguyen AV, Ralainirina N, Hardamon CR, Foubert P, Schmid MC, Sun P, Mose E, Bouvet M (2016). Macrophage PI3Kgamma drives pancreatic ductal adenocarcinoma progression. Cancer Discov.

[32] Carnevalli LS, Taylor MA, King M, Coenen-Stass AML, Hughes AM, Bell S, Proia TA, Wang Y, Ramos-Montoya A, Wali N (2021). Macrophage activation status rather than repolarization is associated with enhanced checkpoint activity in combination with PI3Kgamma inhibition. Mol Cancer Ther.

[33] Sai J, Owens P, Novitskiy SV, Hawkins OE, Vilgelm AE, Yang J, Sobolik T, Lavender N, Johnson AC, McClain C (2017). PI3K inhibition reduces mammary tumor growth and facilitates antitumor immunity and anti-PD1 responses. Clin Cancer Res.

[34] Markham A (2017). Copanlisib: first global approval. Drugs.

[35] Bankhead P, Loughrey MB, Fernandez JA, Dombrowski Y, McArt DG, Dunne PD, McQuaid S, Gray RT, Murray LJ, Coleman HG (2017). QuPath: open source software for digital pathology image analysis. Sci Rep.

[36] Ohue Y, Nishikawa H (2019). Regulatory T (Treg) cells in cancer: Can treg cells be a new therapeutic target?. Cancer Sci.

[37] Patnaik A, Appleman LJ, Tolcher AW, Papadopoulos KP, Beeram M, Rasco DW, Weiss GJ, Sachdev JC, Chadha M, Fulk M (2016). First-in-human phase I study of copanlisib (BAY 80–6946), an intravenous pan-class I phosphatidylinositol 3-kinase inhibitor, in patients with advanced solid tumors and non-Hodgkin's lymphomas. Ann Oncol.

[38] Liu N, Rowley BR, Bull CO, Schneider C, Haegebarth A, Schatz CA, Fracasso PR, Wilkie DP, Hentemann M, Wilhelm SM (2013). BAY 80–6946 is a highly selective intravenous PI3K inhibitor with potent p110alpha and p110delta activities in tumor cell lines and xenograft models. Mol Cancer Ther.

[39] Lechner MG, Karimi SS, Barry-Holson K, Angell TE, Murphy KA, Church CH, Ohlfest JR, Hu P, Epstein AL (2013). Immunogenicity of murine solid tumor models as a defining feature of in vivo behavior and response to immunotherapy. J Immunother.

[40] Chandrasekaran S, Sasaki M, Scharer CD, Kissick HT, Patterson DG, Magliocca KR, Seykora JT, Sapkota B, Gutman DA, Cooper LA (2019). Phosphoinositide 3-kinase signaling can modulate MHC class I and II expression. Mol Cancer Res.

[41] Garcia AJ, Ruscetti M, Arenzana TL, Tran LM, Bianci-Frias D, Sybert E, Priceman SJ, Wu L, Nelson PS, Smale ST (2014). Pten null prostate epithelium promotes localized myeloid-derived suppressor cell expansion and immune suppression during tumor initiation and progression. Mol Cell Biol.

[42] George S, Miao D, Demetri GD, Adeegbe D, Rodig SJ, Shukla S, Lipschitz M, Amin-Mansour A, Raut CP, Carter SL (2017). Loss of PTEN is associated with resistance to anti-PD-1 Checkpoint blockade therapy in metastatic uterine leiomyosarcoma. Immunity.

[43] Liu C, Workman CJ, Vignali DA (2016). Targeting regulatory T cells in tumors. FEBS J.

[44] Qi Z, Xu Z, Zhang L, Zou Y, Li J, Yan W, Li C, Liu N, Wu H (2022). Overcoming resistance to immune checkpoint therapy in PTEN-null prostate cancer by intermittent anti-PI3Kα/β/δ treatment. Nat Commun.

[45] Ostuni R, Kratochvill F, Murray PJ, Natoli G (2015). Macrophages and cancer: from mechanisms to therapeutic implications. Trends Immunol.

[46] Bissell MJ, Hines WC (2011). Why don’t we get more cancer? A proposed role of the microenvironment in restraining cancer progression. Nat Med.

[47] Garrido-Martin EM, Mellows TWP, Clarke J, Ganesan AP, Wood O, Cazaly A, Seumois G, Chee SJ, Alzetani A, King EV (2020). M1(hot) tumor-associated macrophages boost tissue-resident memory T cells infiltration and survival in human lung cancer. J Immunother Cancer.

[48] Jeannin P, Paolini L, Adam C, Delneste Y (2018). The roles of CSFs on the functional polarization of tumor-associated macrophages. FEBS J.

[49] Poh AR, Ernst M (2018). Targeting Macrophages in cancer: from bench to bedside. Front Oncol.

