# Supplementary figures and images for: Pan-PI3K inhibition with copanlisib overcomes Treg- and M2-TAM-mediated immune suppression and promotes anti-tumor immune responses
Source: Clin Exp Med. 2023 Nov 8;23(8):5445–61. doi: 10.1007/s10238-023-01227-6 (PMC10725385; doi:10.1007/s10238-023-01227-6)

Suppl. Fig. 1 (in vitro data)

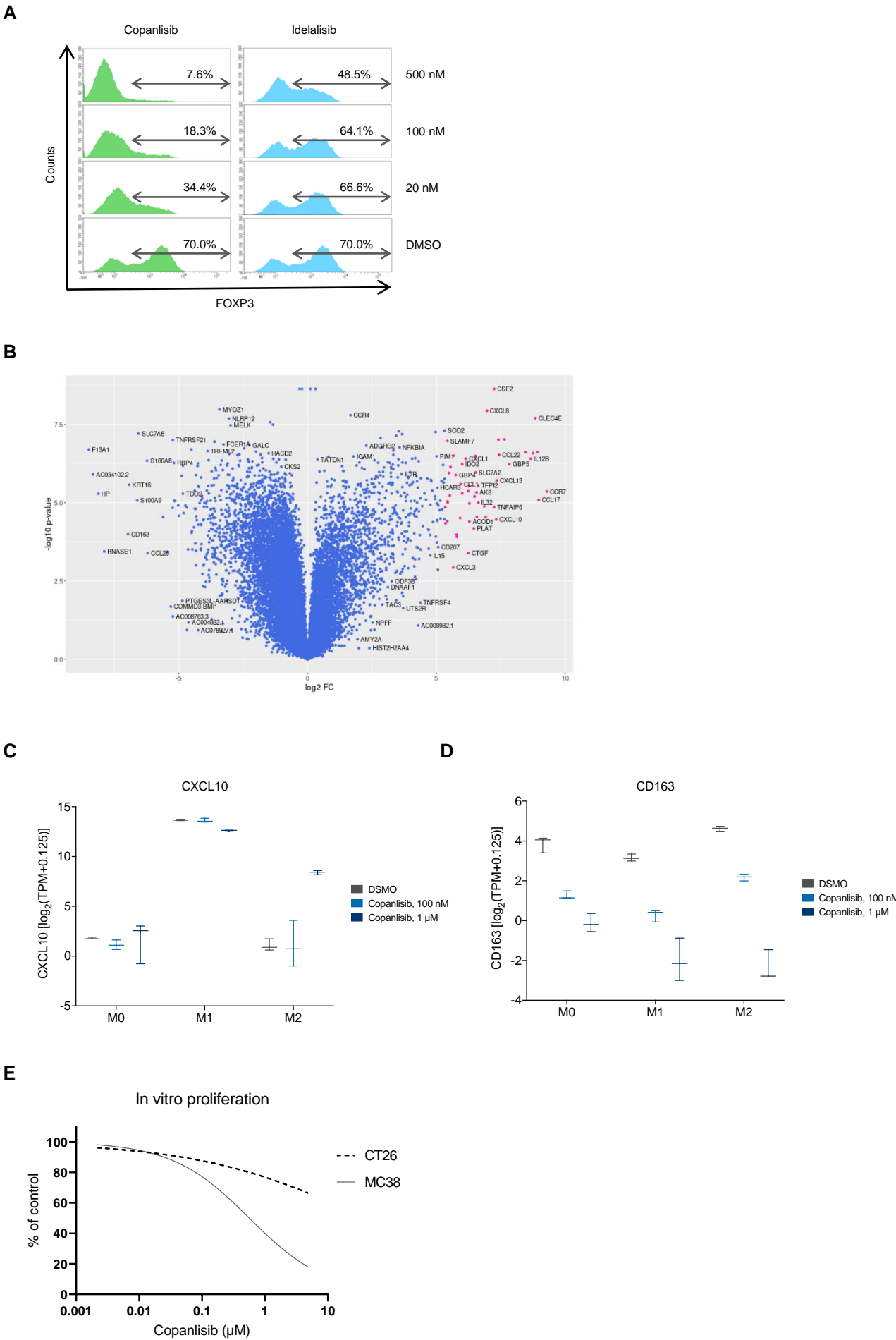

Suppl. Fig. 2 (MC38)

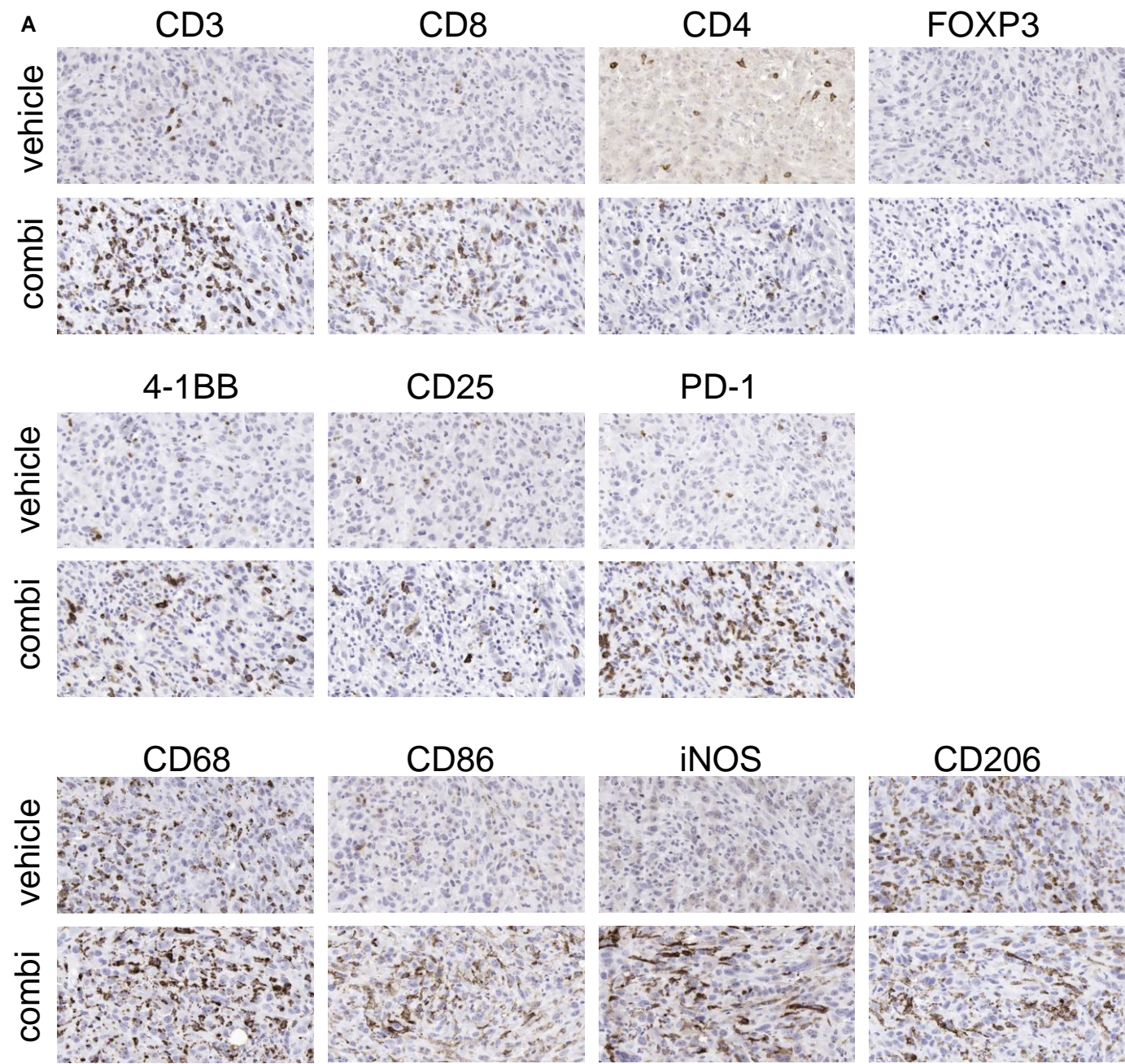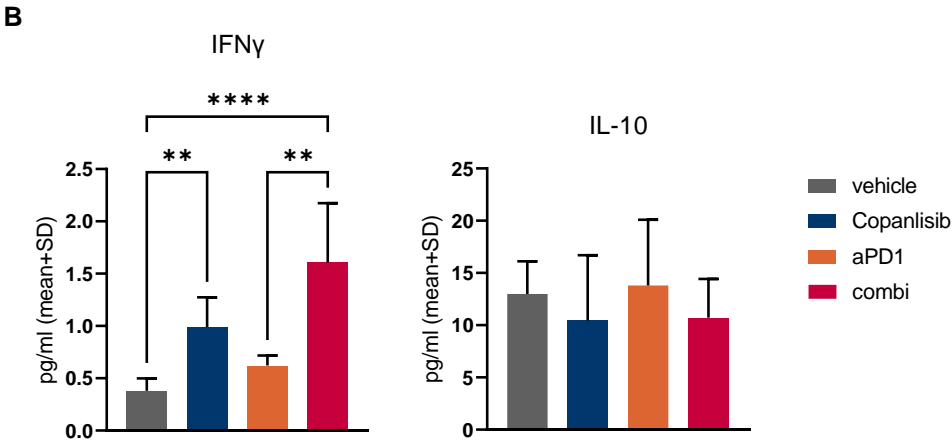

Suppl. Fig. 3 (CT26)

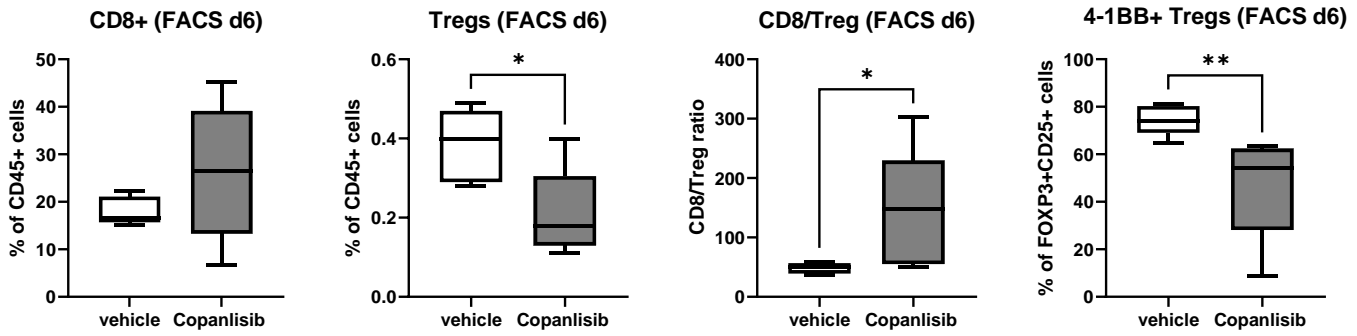

Supplement: Supplementary file 1 — Supplementary file1 (PDF 558 KB) [file 10238_2023_1227_MOESM1_ESM.pdf]
